# Variation and covariation in strongyle infection in East African shorthorn zebu calves

**DOI:** 10.1017/S0031182014001498

**Published:** 2014-09-26

**Authors:** R. Callaby, O. Hanotte, I. Conradie Van Wyk, H. Kiara, P. Toye, M. N. Mbole-Kariuki, A. Jennings, S. M. Thumbi, J. A. W. Coetzer, B. M. de. C. Bronsvoort, S. A. Knott, M. E. J. Woolhouse, L. E. B. Kruuk

**Affiliations:** 1 Institute of Evolutionary Biology, University of Edinburgh, Ashworth Laboratories, Kings Buildings, West Mains Road, Edinburgh EH9 3JT, UK; 2 Centre for Immunity, Infection and Evolution, University of Edinburgh, Ashworth Laboratories, Kings Buildings, West Mains Road, Edinburgh EH9 3JT, UK; 3 James Hutton Institute, Craigiebuckler, Aberdeen AB15 8QH, UK; 4 School of Life Sciences, University of Nottingham, University Park, Nottingham NG7 2RD, UK; 5 Department of Veterinary Tropical Diseases, Faculty of Veterinary Science, University of Pretoria, Private bag X04, Onderstepoort, South Africa; 6 International Livestock Research Institute, P.O. Box 30709, Nairobi 00100, Kenya; 7 African Union – Interafrican Bureau for Animal Resources (AU-IBAR), Kenindia Business Park building, Museum Hill, Westlands Road, P.O Box 30786, 00100 Nairobi, Kenya; 8 The Farm Animal Practice, The University of Edinburgh, Easter Bush, Edinburgh EH25 9RG, UK; 9 The Roslin Institute, Easter Bush, University of Edinburgh, Roslin, Midlothian EH25 9RG, UK; 10 Division of Evolution, Ecology & Genetics, Research School of Biology, The Australian National University, Canberra, ACT 0200, Australia

**Keywords:** gastrointestinal parasite infection, strongyle, indigenous cattle, Kenya, heritability, haematology

## Abstract

Parasite burden varies widely between individuals within a population, and can covary with multiple aspects of individual phenotype. Here we investigate the sources of variation in faecal strongyle eggs counts, and its association with body weight and a suite of haematological measures, in a cohort of indigenous zebu calves in Western Kenya, using relatedness matrices reconstructed from single nucleotide polymorphism (SNP) genotypes. Strongyle egg count was heritable (*h*^2^ = 23·9%, s.e. = 11·8%) and we also found heritability of white blood cell counts (WBC) (*h*^2^ = 27·6%, s.e. = 10·6%). All the traits investigated showed negative phenotypic covariances with strongyle egg count throughout the first year: high worm counts were associated with low values of WBC, red blood cell count, total serum protein and absolute eosinophil count. Furthermore, calf body weight at 1 week old was a significant predictor of strongyle EPG at 16–51 weeks, with smaller calves having a higher strongyle egg count later in life. Our results indicate a genetic basis to strongyle EPG in this population, and also reveal consistently strong negative associations between strongyle infection and other important aspects of the multivariate phenotype.

## Introduction

Gastrointestinal parasite infections of livestock are responsible for large economic losses in pastoral systems (Keyyu *et al.* 2003). They reduce weight gain and fertility, and may even cause direct losses through mortality (Wymann *et al.* 2008). Reduction of gastrointestinal parasite infections would therefore improve animal health and remove some of the constraints on livestock enterprises in developing countries, thereby reducing poverty (Perry and Sones, 2007). However, management of parasite infection requires an understanding of the causes of variation in parasite burdens, variation which can be substantial even between individuals within a population. For example, in indigenous East African Shorthorn Zebu (*Bos indicus*, EASZ) calves in Western Kenya, most individuals experience an apparently low level of strongyle worm infection, whilst others experience a high worm burden and suffer severe consequences (Thumbi *et al.* 2013a). In this paper, we explore possible causes of this variation, and quantify its covariation with other variables.

Strongyles are a group of nematode gut worms which produce morphologically similar eggs. Strongyle-type eggs include the species *Haemonchus placei, Trichostrongylus axei* and *Oesophagostomum radiatum* (Urquhart *et al.* 1996). The most common method used to quantify worm burden is a count of the number of strongyle eggs per gramme of faeces (EPG), a non-invasive, relatively easily measured variable. It has been shown that faecal egg counts (FECs) are a good index of parasite burden in Australian cattle, although the relationship between the two may not be exactly linear (Bryan and Kerr, 1989). Variation in strongyle FEC can be due to a variation in susceptibility, resistance, tolerance or exposure to infection by strongyle worms. Evidence from other domestic ungulates suggests that variation in strongyle FEC frequently has a heritable genetic basis: for example, FEC has a heritability of 18% (95% CI = 0·10–0·25) in West African N’Dama cattle (Zinsstag *et al.* 2000), and the heritability is approximately 30% in many other cattle breeds (Stear *et al.* 1988, 1990; Leighton *et al.* 1989). Similarly, strongyle EPG in Scottish Blackface sheep lambs has a heritability of 32% (Riggio *et al*. (2013); see also Beraldi *et al*. (2007); Crawford *et al*. (2006)).

In addition to additive genetic effects, there may also be consistent environmental-based causes of variation in parasite burden between individuals. These ‘permanent environmental effects’ comprise all variance of non-(additive) genetic origin that persist throughout an individual’s life-time, and so for example may include long-running effects of maternal environment or of how an individual was raised and housed: for instance, in a feral Soay sheep population, lambs born as twins or born to very young or old mothers have higher parasite burdens than those born as singletons or to prime-age mothers (Hayward *et al.* 2010). Stear *et al.* (1996) also found higher parasite burdens in Scottish Blackface sheep twins in comparison to singletons. The physical environment that an individual resides in will also be important for determining its exposure to a particular pathogen, which in turn can affect the burden of infection observed (e.g. Batchelor *et al.* 2009). Finally, there may be variation between measures made on an individual at different time points, due to, for example, effects of ageing, immediate climatic effects or simply stochastic variation and measurement error.

Variation in parasite burden may also have implications for the expression of other important traits, especially if parasite resistance is costly and may therefore be traded off against investment in other traits (Norris and Evans, 2000). Such associations can be quantified within individuals by looking at the covariation of parasite burden and other traits, for example morphological variables such as growth rates or weight, or physiological variables, such as haematological parameters, to test for any costs associated with high parasite burdens (e.g. Coltman *et al.* 2001). In particular, one of the strongyle species, *H. placei,* is an important cause of anaemia in ruminants (Kaufmann *et al.* 1992): Conradie van Wyk *et al.* (2013) and Vanimisetti *et al.* (2004) have shown negative correlations between parasite burden and various haematological parameters in EASZ and sheep. Finally, it is possible that an individual’s phenotype at birth may influence their infection risk later in life. For example, in humans, babies that have a lower birth weight are more likely to develop lower respiratory tract infections when they are coinfected with hand, foot and mouth disease (Lu *et al.* 2013). Likewise, Read *et al.* (1994) showed there is a higher risk of childhood infectious disease mortality in lower birth weight babies than heavier ones.

Traditionally, pedigree information has been used to estimate quantitative genetic parameters such as the heritability of a trait (Falconer and Mackay, 1996). More recently, the development of high density SNP beadchips means that novel alternative approaches can be used without reference to pedigree records (Yang *et al.* 2010; Visscher *et al.* 2014). This has reduced previous constraints faced during estimation of heritability in wild populations due to the lack of accuracy and completeness of the pedigree (Pemberton 2008). Bérénos *et al.* (2014) compared heritability estimates produced from using both pedigrees and SNPs from related Soay sheep and demonstrated that heritability estimates obtained from dense SNP data are in correspondence with pedigree estimates.

The Infectious Diseases of East African Livestock (IDEAL) project (Bronsvoort *et al.* 2013) provides a unique opportunity to study natural variation and covariation in strongyle EPG in indigenous EASZ from Western Kenya. Cattle in this region are minimally managed and there is very limited use of vaccination or other preventative measures against infectious diseases. Therefore the study population is similar to a wild population in that, unlike other estimates of genetic variation in FEC in domestic populations (e.g. Bishop *et al.* (1996)), animals have not been treated for anthelmintics (those individuals which were treated with anthelmintics were retrospectively removed from the cohort as part of the IDEAL study design); variation therefore reflects natural diversity in parasite burden. Calves were enrolled in the study at birth and their infectious disease burden, haematological profiles and growth were tracked for the first year of life (Conradie van Wyk *et al.* 2012; Bronsvoort *et al.* 2013). Strongyle worm burdens (assessed via EPG) have a major impact upon the calves in the study population: for example, an increase in strongyle EPG by a count of 1000 eggs is associated with a 3·3% reduction in weight gain over the first year (Thumbi *et al.* 2013b), and an increase in the hazard of death by 1·5 (95% CI = 1·4–1·7, *P* < 0·001; Thumbi *et al*. (2013a)). Moreover, genome-wide genetic information is available in the form of SNPs as each calf enrolled in the IDEAL project was genotyped with a 50 K Illumina^®^ BovineSNP50 beadchip (Murray *et al.* 2013; Mbole-Kariuki *et al.* 2014), providing the opportunity to exploit this information to estimate a relatedness matrix and thereby derive estimates of variance components, including additive genetic variance of different traits.

Our aim in this study is to dissect the potential genetic and non-genetic sources of between- and within-individual level variation in strongyle EPG. We present a multivariate analysis of associations between strongyle EPG, body size and a suite of haematological measures. We quantified the variance components of five physiological traits and their covariation with strongyle EPG. Finally we investigated whether the characteristics of newborn calves could be used to predict subsequent EPG levels, by looking at the association between weight at birth and strongyle EPG later in life.

## Materials and Methods

### Study population

Five hundred and forty-eight free-grazing indigenous EASZ calves in Western Kenya were selected using a stratified two-stage random cluster study design. In the first stage, 20 sublocations (the smallest administrative unit in Kenya) were selected from five agro-ecological zones, across an area of roughly 45 × 90 km. Around 28 3–7-day-old calves were recruited from each sublocation, all from different mothers and different farms; see Bronsvoort *et al.* (2013) for a detailed description of the study design. Recruited calves were followed for their first year of life. They were visited every 5 weeks for a clinical examination at which they were weighed and blood and faecal samples were taken for parasite identification and haematological profiling. A total of 446 calves that survived to 51 weeks of age (and had passed the SNP quality control checks, see SNP quality control section below) were included in this analysis, giving a total of 4727 observations and an average of 10·6 visits per calf.

### Data collection

The McMaster counting technique (Hansen and Perry, 1994) was performed on the faecal samples from each visit to each calf to quantify the number of strongyle eggs per gramme of faeces (EPG) present. We refer to our measurement of strongyle faecal egg count as EPG (eggs per gramme); though note that this may also be referred to as FEC in the literature.

The other traits considered in this study were: white blood cell count (WBC), red blood cell count (RBC), total serum protein (TSP), absolute eosinophil count (EO) and body weight. Blood cell analysis was automatically performed using the pocH-100iV Diff (Sysmex® Europe GMBG); see Conradie van Wyk *et al.* (2012) for more details. Haematological profiles were produced for the total WBC and RBC. TSP was determined using a refractometer and EO was quantified by differential counts from thin EDTA blood smears stained with Diff Quick. Previous studies have shown that higher RBC and heavier body weights are associated with lower FECs (Conradie van Wyk *et al.* 2013; Thumbi *et al.* 2013b; Vanimisetti *et al.* 2004).

Calves were weighed (in kilogrammes, measured to the nearest 500 g) at recruitment, then again every 5 weeks until 31 weeks of age, and once again at a last visit at 51 weeks. The number of observations for each trait is presented in Table 1.

### SNP quality control and construction of the relationship matrix

All calves were genotyped using a 50 K Illumina^®^ BovineSNP50 beadchip v.1. The beadchip contained 55 777 SNPs before quality control, spread evenly throughout the genome with an average of 1895 SNPs on each autosome and 1362 SNPs on the X chromosome (Murray *et al.* 2013). Quality control was applied to all SNP data prior to analysis using GenABEL (Aulchenko *et al.* 2007), with the following criteria: SNP call rate cut-off of 0·9; individual call rate of 0·9 and an identity by state (IBS) threshold cut-off of 0·9. The IBS threshold means that if a pair of individuals is estimated to be exceptionally highly related (e.g. identical twins) then one of the individuals would be removed. The minimum minor allele frequency for SNPs was set to 0·005, to include all SNPs with a minor allele count of 5 or more. Any X chromosome genotypes that were inconsistent with the phenotype were removed. This quality control resulted in 42119 autosomal and X markers (41 419 autosomal markers plus 700 X markers) and 446 calves for analysis. We explored the effect of varying the quality control parameters and the number of SNPs included in the IBS matrix on the resulting estimates of heritability; details are given in Supplementary Tables 1 and 2; in general, estimates of heritability for strongyle EPG increased with increasing marker density. Plots of the distribution of the minor allele frequencies at SNP markers and the association between linkage disequilibrium and the distance between pairs of SNPs are presented in Supplementary Figure 1.

All SNPs and calves which passed the quality control checks were then used to construct an identity-by-state matrix in GenABEL (Aulchenko *et al.* 2007) using the allele frequency weighted option, giving the kinship coefficients for use in the variance component and heritability analyses described below. The average genomic estimate of kinship between calves as given by the IBS matrix ranged from −0·02 to 0·24. Three pairs of calves had a genomic estimate of relatedness greater than 0·2 and 6 pairs of calves had a genomic estimate of relatedness between 0·15 and 0·2.

Approximately 20% of the calves in the IDEAL study cohort were shown to have some level of introgression from European taurine (ET) cattle, although calves that were first generation offspring from ET were explicitly excluded from the study (Bronsvoort *et al.* 2013; Mbole-Kariuki *et al.* 2014). These calves with lower levels of ET introgression were included in our study since the aim of the study was to describe the components of variation in strongyle EPG in the population. The effect of excluding the introgressed calves on the heritability estimates is presented in the supplementary materials (Supplementary Table 3).

### Statistical analysis

#### Trait distributions

In order to account for the distribution of the strongyle EPG counts, we used generalized linear mixed models (GLMMs) with a negative binomial distribution and log link function; as observations of strongyle EPG were in multiples of 50, they were first divided by 50 so that the data resemble typical count data. Note that estimates of variance components for EPG are therefore on a latent scale rather than on the original data scale (Nakagawa and Schielzeth, 2010). All other variables were analysed assuming Gaussian distributions. Body weight was first transformed to log_10_ (weight) and EO to log_10_ (EO + 1) to account for their slightly skewed distribution.

A significant increase in RBC was found between the calves aged 1 *vs* aged 6 weeks old, followed by a general decreasing trend in calves aged 6–51 weeks (Supplementary Figure 2 and Conradie van Wyk *et al.* 2012). We therefore focused our analysis of RBC on calves aged 6–51 weeks old for RBC. Removal of the records from 1-week-old calves did not affect the direction of associations observed and only resulted in small changes to the variance and heritability estimates.

#### Random effects and variance components estimation

We used an animal model to estimate the variance components of each trait (Lynch and Walsh, 1998; Kruuk 2004). Animal models are a form of mixed model, with fixed and random effects, that can break phenotypic variation down into the different components via a model of the form:

y=Xb+Za+Pc+Sd+e

where ***y*** is the phenotype of interest and ***b*** is a vector of fixed effects that are unknown constants that affect the mean of the distribution. The random effects, which determine the variance of the trait, were additive genetic (***a***), permanent environment (***c***), sublocation (***d***) and residual effects (***e***). In particular, ***a*** is a vector associated with the identity-by-state matrix (see Visscher *et al.* (2008) and Powell *et al.* (2010) for more details on calculating heritabilities using identity-by-state matrices rather than pedigrees) and is derived from the principle that if a trait has a high degree of genetic variance relative to its other components of variance, pairs of relatives will have high phenotypic similarity. ***X***, ***Z***, ***P*** and ***S*** are all design matrixes corresponding to the appropriate fixed or random effects. Permanent environmental effects are measurable because of the repeated observations on the same individual; this between-individual variation is likely to result from long-term environmental or non-additive genetic effects, and in this case will probably incorporate most of any maternal effects (Kruuk and Hadfield, 2007). The total phenotypic variance (*V_P_*) for a trait was therefore broken down into the additive genetic variance (*V_A_*), permanent environmental variance (*V_PE_*), sublocation variance (*V_SL_*) and residual variance (*V_R_*):

$$V_{P}=V_{A}+V_{PE}+V_{SL}+V_{R}$$

The narrow-sense heritability of a trait (*h^2^*) is defined as the proportion of phenotypic variance (*V_P_*) explained by the additive genetic variance (*V_A_*), *h^2^* = *V_A_/V_P_.* It describes the extent to which differences between individuals are determined by additive genetic effects (Falconer and Mackay, 1996). We also report the repeatability (*r*^2^) of each trait, defined as the proportion of the phenotypic variance due to consistent differences between individuals and is given by the ratio of the between individual variance to the total variance, *r*^2^ = (*V_A_* + *V_PE_* + *V_SL_*)/*V_P_*.

The covariances between traits can be investigated using multivariate models. By extending the above approach of variance partitioning to multiple traits, and linking them through a covariance term in the random effects, we can ask how much of the phenotypic covariance (*COV_P_*) between traits is due to covariance of the different random effects described above, for example covariance in the permanent environment effects (*COV_PE_*).

All statistical analyses were carried out in ASReml version 3.0.5 (Gilmore *et al.* 2006).

#### Components of variation in strongyle EPG

Estimation of the components of variance of strongyle EPG at each visit indicated that there was insufficient statistical power to analyse measures at every visit separately. In order to overcome this, we used a univariate animal model fitted with a negative binomial distribution to estimate the heritability of strongyle EPG across all ages. Age (as a multi-level factor) was fitted as a fixed effect to account for changes across visits in mean EPG with age. Sex was also included in this model as a fixed effect and *V_A_*, *V_PE_* and *V_SL_* were fitted as random effects. Unlike other studies which have estimated genetic variation in FEC in domestic animals (e.g. Bishop *et al.* (1996)), individuals in this study population have not been treated with anthelmintics, and so represent natural levels of variation. Repeated observations on individuals are therefore not necessarily independent assessments of resistance, because nematodes might persist between sample dates. However our mixed models account for the repeated measures structure of the data by fitting a permanent environment effect, defining the number of individuals as the appropriate number of independent observations (Kruuk and Hadfield 2007).

The significance of *V_A_* was evaluated by comparing the component estimate to the standard error, as it is not advisable to carry out likelihood ratio tests (LRTs) for GLMMs with negative binomial errors in ASReml (Gilmore *et al.* 2006). Finally, for comparison with previous studies which have analysed FECs assuming Gaussian errors (Stear *et al.* 1990; Bishop *et al.* 1996; Coltman *et al.* 2001; Beraldi *et al.* 2007), we also present analyses of linear mixed models assuming a normal distribution of log_10_ (strongyle EPG + 50). These results are presented in the supplementary materials.

#### Components of variation in physiological traits

The components of variance in the physiological traits were examined by constructing a univariate Gaussian repeated measures animal model for each trait. As above, age and sex were included as fixed effects, and *V_A_*, *V_PE_* and *V_SL_* were fitted as random effects in all models. The significance of *V_A_* for each trait was assessed with a LRT comparing the full animal model to one in which the additive genetic variance was set to zero.

#### Associations between strongyle infection and physiological traits

We assessed associations between strongyle infection and the physiological traits (and body size) in three different ways, by: (1) testing whether infection affected mean levels of the physiological traits; (2) testing whether size at birth predicted levels of strongyle infection later in life; and (3) assessing components of covariance between all traits.

The effects of strongyle infection on the physiological traits were therefore first quantified by univariate animal models with the trait as the response variable and explanatory variables of age at visit, calf sex and strongyle EPG classified into two categories of ‘high’ and ‘low’ EPG. A ‘high’ strongyle EPG was defined as a value above the median strongyle EPG across all visits (200 EPG), and a ‘low’ strongyle EPG one below the median. This categorization was chosen to reflect the non-linearity in effect of strongyle EPG estimate of effect (Conradie van Wyk *et al.* 2013). All of the explanatory variables were coded as factors and *V_A_*, *V_PE_* and *V_SL_* were fitted as random effects.

**Fig. 1:**
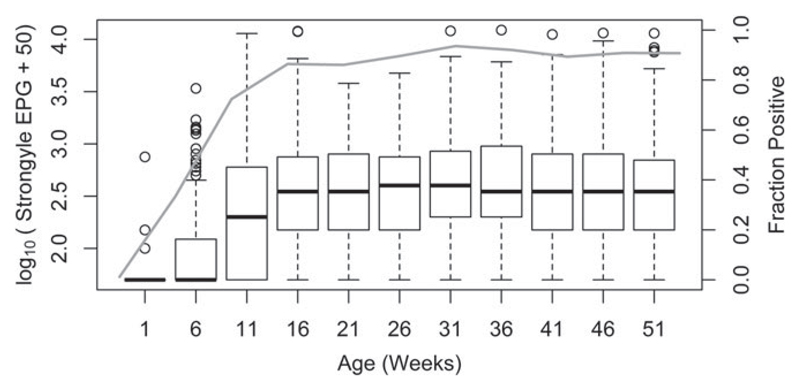
Distribution of strongyle EPG (box plots, left-hand axis) and the fraction of calves which tested positive at each age (right-hand axis). The black heavy solid lines in each box are the median EPG at each age group, the bottom and top of the box represent the 25^th^ and 95^th^ percentiles, respectively, and the whiskers represent 1·5 times the interquartile range. Points beyond the whiskers are outliers. Strongyle EPG is transformed as log_10_(strongyle EPG + 50). The solid grey line represents the fraction of tested calves positive for strongyle eggs at each age.

Secondly, we tested whether a calf’s phenotype very early in life was an informative predictor of our index of infection burden, EPG, later in life, and specifically whether the calf’s recruitment weight predicted strongyle EPG later in the first year of life. This was achieved by constructing a univariate animal model with a negative binomial distribution to evaluate the effect of calf weight at recruitment (when the calf is less than 1 week old) on strongyle EPG in older calves (aged 16–51 weeks, following a plateau in median strongyle EPG after 16 weeks, Figure 1). This model includes calf age and sex as fixed effects and *V_A_*, *V_PE_* and *V_SL_* as random effects. The magnitude and directionality of association between the trait and strongyle EPG is given by the parameter estimate, whilst its significance was assessed using Wald F statistics.

Thirdly, the covariances and correlations between strongyle EPG and the physiological traits were assessed by constructing a multivariate model of all six traits (strongyle EPG, WBC, RBC, TSP, EO and weight), using measures across the whole year for all traits. Calf age and sex were included as fixed effects and strongyle EPG was fitted with a negative binomial error distribution, whilst the other traits were fitted with a Gaussian error distribution. The resulting six-trait multivariate model was computationally much more demanding than the univariate models described above, due to the much greater number of parameters (an extra 80 parameters) being estimated. We therefore had to take several steps to facilitate reliable convergence. Firstly, we were unable to separate between-individual differences into genetic *vs* permanent environment effects, so we restricted the analysis to separating between- *vs* within-individual-level variances and covariances, omitting the genetic relationship matrix from the model. By only including calf identity as a random effect, we obtained estimates of the individual- (phenotypic-) level variance, which reflects consistent differences between individuals; similarly, the model partitions the total phenotypic *covariance* between two traits into that due to between-individual *vs* within-individual (residual) components. Secondly, we were unable to fit sublocation as a random effect in the models, so it was omitted from the multivariate analysis. Note however that sublocation was never significant in any of the univariate models (Table 1), and its effects will be included in the permanent environment effect (co)variance. Since LRTs are not advisable with GLMMs with negative binomial errors in ASReml (Gilmore *et al*. 2006), significance of estimates was assessed based on their magnitude relative to the standard error.

## Results

### Summary statistics

Out of the 4032 visits with faecal samples taken from the 446 live calves that passed the genetic quality control checks, strongyle eggs were detected in 3071 (76·2%) visits using the McMasters technique. The overall median number of strongyle EPG of faeces was 200 EPG (range: 0–12250 EPG). All calves were infected with strongyle eggs at some point during their 51 weeks of inclusion in the study. Infection rates increased up to 16 weeks of age, and then levelled off afterwards, with an average of 89·8% of visits showing non-zero EPG between the ages of 16–51 weeks, and a median strongyle EPG of 300 EPG (range: 0–12250 EPG). The median strongyle EPG and the fraction of calves positive at each age are shown in Figure 1.

### Components of variation in strongyle EPG

**Fig. 2:**
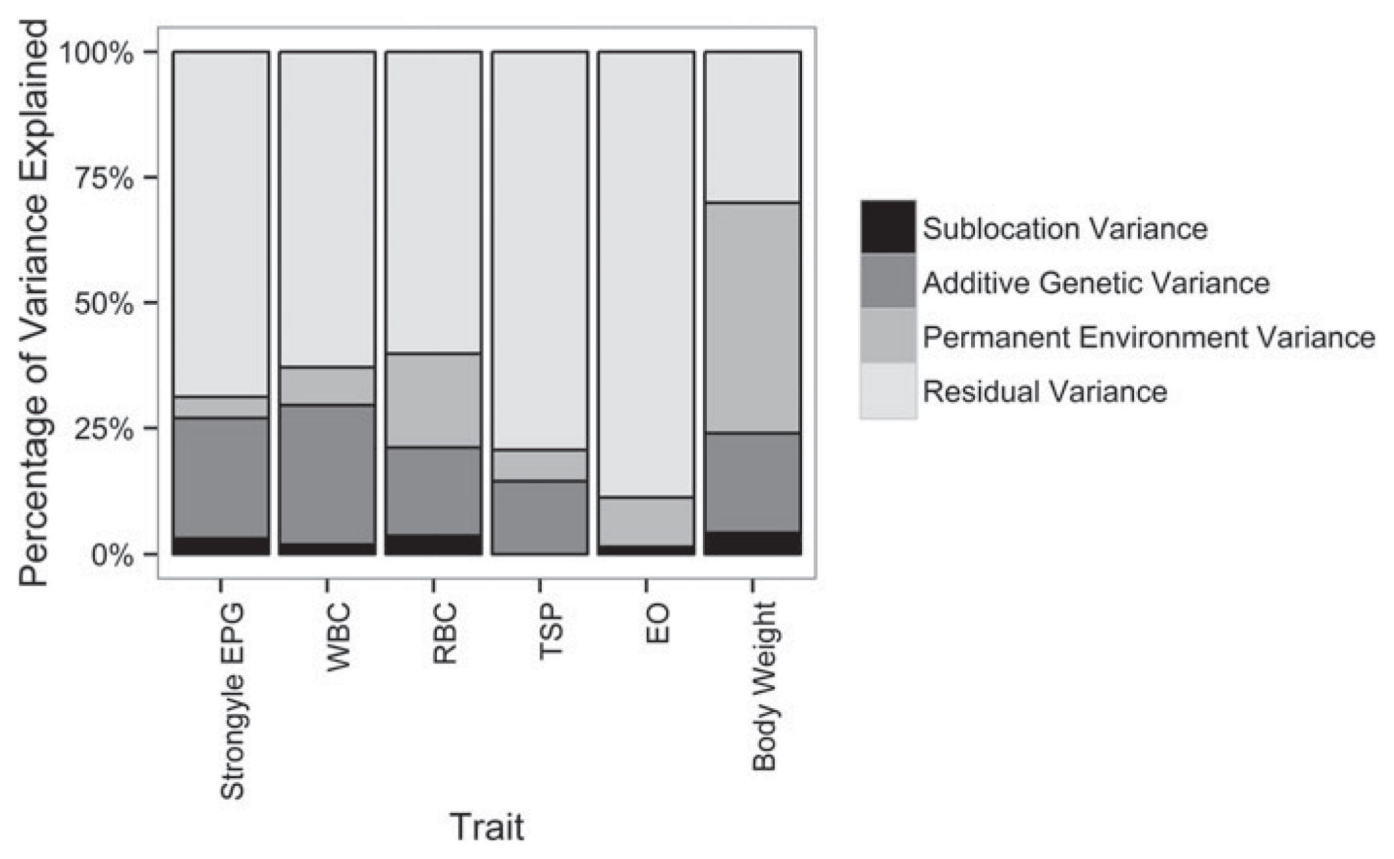
Percentage of variance explained by each component for all traits investigated. Details of the full model (including SEs on variance component estimates) are given in Table 1. Strongyle EPG uses an animal model fitted with a negative binomial distribution model. WBC, white blood cell count (× 10^3^*μ*L^−1^); RBC, red blood cell count (× 10^6^*μ*L^−1^); TSP, total serum protein (g dL^−1^); EO, transformed absolute eosinophil count (× 10^3^ *μ*L^−1^, log_10_(EO + 1)); body weight, transformed body weight (kg, log_10_(weight)).

Additive genetic variance contributed the most (after residual variance) to the overall variance in strongyle EPG, resulting in heritable variation in strongyle EPG in EASZ calves (*h*^2^ = 23·9%, s.e. = 11·8%, Table 1). In contrast, the contribution of permanent environmental effects to the overall variance was relatively low (4·3%, s.e. = 11·5%, Table 1 and Figure 2). Strongyle EPG had a repeatability of 31·4% (s.e. = 2·2%). In addition, male calves had a higher strongyle EPG than female calves (effect estimate = 0·23, s.e. = 0·08, *P* value = 0·01).

Complete removal of the ‘introgressed’ calves from the study resulted in a lower heritability estimate and larger standard errors, whilst inclusion of the ET introgression as a fixed effect did not alter the heritability estimate (with ET introgressed calves included *h*^2^ = 23·9%, s.e. = 11·8%, *N* calves = 446; with ET introgression included as a fixed effect, *h*^2^ = 25·7%, s.e. = 11·9%, *N* calves = 446; with ET introgressed calves excluded *h*^2^ = 13·3%, s.e. = 13·4%, *N* calves = 353; see Supplementary Tables 1 and 3).

For comparison of the negative binomial errors model with models assuming Gaussian errors, we present analyses of linear mixed models assuming a normal distribution of log_10_ (strongyle EPG + 50) in Supplementary Table 4. Both methods produce similar estimates of heritability of strongyle EPG although, notably, the standard errors are much larger with the GLMM.

### Components of variation in physiological traits

The age-related profiles for the physiological traits are shown in Supplementary Figure 2 (split according to whether the calf had high or low EPG at the time). WBC, EO and weight all increased with age, as expected. However, RBC increased rapidly until 6 weeks old and then declined sharply. A decline from birth in TSP was observed until 21 weeks of age when TSP started to increase again. These distributions and the effect of coinfections on WBC are discussed in Conradie van Wyk *et al*. (2012) and (2013), respectively.

Estimates of the variance components and the heritability of each trait are shown in Table 1. WBC was the only physiological trait to show evidence for significant *V_A_* (LRT: *χ*^2^ = 8·8, d.f. = 1, *P* = 0·003, Table 1; *h*^2^ = 27·6%, s.e. = 10·6%). There were large differences between traits in the proportion of the total variance (*V_P_*) explained by each variance component: for example, permanent environment effects explained most (45·9%, s.e. = 19·1%) of the total variance in body weight, but only a relatively small proportion of the variance in the other parameters (7·1–18·5%; Table 1). Weight had the highest repeatability of 69·9% (s.e. = 1·7%); repeatability otherwise ranged from 11·3% (s.e. = 1·4%) for EO to 37·2% (s.e. = 2·0%) for WBC.

### Associations between strongyle infection and physiological traits

#### Effect of strongyle infection on physiological traits

We found significant effects of strongyle infection on all the physiological traits considered. The impact of strongyle EPG on every trait at each age is illustrated in Supplementary Figure 2 and quantified in Table 2. Table 2 shows that calves with a higher strongyle EPG at a given age tended to have a lower RBC, TSP and EO than those with a lower strongyle EPG. Furthermore, calves with a high strongyle EPG were also lighter than those with a lower EPG (by −0·02 log_10_ (kg); s.e. = 0·03 on average, Table 2). Similar results were observed when a continuous measure of EPG (log_10_ (strongyleEPG + 50)) rather than a binary measure was used as an explanatory variable.

#### Does weight at first visit predict strongyle infection in older calves?

Weight at the recruitment visit (when the calf was less than a week old) was significantly associated with later strongyle EPG: calves that were lighter at the first visit had a higher strongyle EPG when aged 16–51 weeks old than calves that were heavier (Table 3). As above, males also had higher levels of EPG.

#### Covariances between strongyle EPG and physiological traits

The individual-level and residual covariances between strongyle EPG and the physiological traits of interest are shown in Table 4. All traits had a negative individual-level covariance with strongyle EPG whilst positive covariances were found amongst all the blood parameters and weight. This indicates that an increase in strongyle EPG was associated with a decrease in blood parameters and weight, whilst an increase in weight, etc. was associated with an increase in blood parameters and *vice versa*. Comparison of the between-individual *vs* residual (within-individual) variance showed that both follow the same pattern, but that there were higher levels of between-individual than residual (within-individual) level correlations.

## Discussion

Our analyses of data from zebu calves in Western Kenya quantified several sources of variation: firstly, in strongyle worm burdens, and secondly in body size and a suite of haematological parameters that we anticipated might be affected by strongyle infection. Measures of associations between strongyle EPG and the physiological traits were consistently negative, suggesting a possible cost of increased parasite burdens. Below, we discuss each of these aspects of our results in turn.

### Components of variation in strongyle EPG

Our results show, firstly, substantial changes with age in median levels of strongyle EPG in EASZ calves. The difference in median strongyle EPG in young (age 1–11 weeks) and old (age 16–51 week) calves is possibly due to weaning, with calves moving more once they are weaned and so older calves being at higher risk of becoming infected due to sampling more areas. We observe lower median FECs then might normally be expected for *Haemonchus* infections (e.g. compare to Hansen and Perry (1994)). However, Kanyari *et al.*’s (2010) study of cattle from a peri-urban area in a neighbouring area of Western Kenya (which included exotic breeds) observed a similar prevalence and mean strongyle EPG (mean = 296, range = 0–8300 EPG (Kanyari *et al.* 2010) and Fig. 1 for comparison). Secondly, as in other studies for example (Hayward *et al.* (2009) and Moore and Wilson (2002)); male calves have a higher strongyle EPG then female calves.

Thirdly, our analyses indicated that strongyle EPG was heritable (*h*^2^ = 23·9%, s.e. = 11·8%). Similar heritabilities have been observed in feral Soay sheep lambs on St Kilda (*h*^2^ = 26%, s.e. = 12%, Beraldi *et al*. (2007)) and in Scottish Blackface sheep ewes (*h*^2^ = 23%, s.e. = 9%, (Bishop and Stear, 2001)). These estimates are from models which included the same fixed effects of age and sex as used in our analysis, but they also included additional fixed effects such as weight and twin status, so direct comparisons of heritability need to treated cautiously (Wilson, 2008). As we have found evidence for the presence of heritable variation in strongyle EPG, it may therefore be possible for selection for parasite resistance to occur. Quantitative trait loci and SNPs associated with strongyle FEC have been identified in Soay sheep (Beraldi *et al.* 2007) and Blackface lambs (Riggio *et al.* 2013), but so far have not yet been tested for in indigenous African cattle.

Lastly, complete removal of the ‘introgressed’ calves from the study resulted in a lower heritability estimate and larger standard errors. The decrease in heritability is possibly due to European introgressed calves having a higher genetic variance whilst the larger standards errors are likely to be due to a decrease in sample size. Inclusion of the ET introgression as a fixed effect did not alter the heritability estimate. However, as the focus of the aim of this study is to describe the components of variation in strongyle EPG in the study cohort we wish to include as much variance in the population as possible in the dataset. Furthermore, the level of ET introgression is on a continuous scale and the cut-off to determine what level of introgression should be excluded is somewhat arbitrary.

### Components of variation in physiological traits

We found evidence for significant additive genetic variation in WBC in our study population (WBC, *h*^2^ = 27·6%, s.e. = 10·6%; *V_A_* = 3·1, s.e. = 1·2). This is in accordance with other analyses of WBC count, which have found it to be heritable in both humans and pigs (*h*^2^ = 35%, s.e. = 9%, Pankow *et al.* (2001); *h*^2^ = 29%, s.e. = 10%, Clapperton *et al.* (2009), respectively). None of the other traits investigated showed evidence for significant additive genetic variation. However, Rowlands *et al.* (1995) showed that packed red-cell volume was heritable in zebu (*h*^2^ = 32%, s.e. = 7%, sample size = 936) and body weight is known to be highly heritable in many other species, including in a much larger study of beef cattle (*h*^2^ = 41%; Marshall (1994)). More generally, haematological parameters are highly heritable in humans, for example haemoglobin levels, RBC, WBC and platelet numbers have heritability estimates of 37, 42, 62 and 57%, respectively (Garner *et al.* 2000). The difference with our results may reflect limited statistical power. In addition, age may be playing an important role in determining the overall (co)variance seen, as heritability (of for example weight and hindleg length in Soay sheep) changes with age (Wilson *et al.* 2007). Furthermore, all of these traits are likely to be polygenic, and so are influenced by many loci of small effect (Goddard and Hayes, 2009), and so it is unlikely that all of the causal loci were detected given the low linkage disequilibrium in EASZ (see below).

### Possible biases in heritability estimation

Using our SNP data, we have demonstrated here that it is possible to estimate the heritability of select traits without the need for pedigree information or even the presence of close relatives. We found evidence of heritable variation in strongyle EPG and in WBC. However, it is worth noting that our estimates may be slightly lower than the true heritability because of the ascertainment bias of the SNP chip (Matukumalli *et al.* 2009). Additionally, in the absence of close relatives (such as in our study sample, as all the calves had different mothers and the average genomic relatedness from the IBS matrix ranged from −0·02 to 0·24, and only 9 pairs of calves out of the 446 individuals had a genomic estimate of relatedness greater than 0·15), the heritability estimated is determined by the variance explained by causal variants that are in linkage disequilibrium with the genotyped SNPs (Yang *et al.* 2010). Mbole-Kariuki *et al.* (2014) showed that EASZ have lower levels of average linkage disequilibrium between adjacent SNP pairs on the SNP chip than other cattle breeds (Nelore and N’dama cattle). Therefore the residual relatedness (i.e. between two ‘unrelated’ individuals) is low; consequently unrelated individuals (by known pedigree) will only share very short proportions of the genome. Furthermore, as marker density increases, our estimate of heritability also increased (Supplementary Table 2). These factors suggest that our estimates of heritability may be lower than those which would be estimated using more closely related individuals and more dense markers (Yang *et al.* 2011; Bérénos *et al.* 2014). Similarly, Robinson *et al.* (2013) found marker-based estimates to be as low as 60% of the value of pedigree-based estimates of heritability of wing length in a wild bird population.

As such, the estimates presented here should be taken as lower limits on the true estimates of heritability of the different traits in this population, which may also explain why we did not find significant heritability for body weight (*h*^2^ = 19·6% s.e. = 19·2%), a trait which is commonly found to have significant additive variance. However, conversely, use of known relatives can result in an overestimation of the true heritability as relatives may share non-additive effects such as dominance, epistasis and shared environmental conditions, which may then confound estimates of similarity due to genetic effects if not adequately accounted for (Kruuk and Hadfield, 2007). Since our study does not include close relatives, our estimates will not be affected by this issue.

Care needs to be taken in distinguishing additive genetic effects from other sources of variance in this analysis as maternal or shared environment effects may be important. The IDEAL dataset has information on only one calf per mother; therefore we cannot estimate maternal effects explicitly. However, this data structure also means that maternal effects are less likely to confound estimates of additive genetic variance, as the most usual scenario is that covariance between full-sibs or maternal half-sibs due to maternal effects is mistaken for additive genetic effects (Kruuk and Hadfield, 2007). Any maternal effects are most likely to be contained in the permanent environment effect variance; however, there is also the possibility that if the maternal effects themselves are to any extent genetically based and if related mothers are in the same sublocation, they may also contribute to the sublocation variance. Note however that all calves were from different farms, so very immediate local effects will not be generating any covariance between individuals.

It is also worth pointing out that our estimates had relatively large standard errors, especially for the parameters associated with additive genetic effects. This may be a result of the relatively small sample size (446 individuals) and a lack of relatedness structure between calves, though our sample sizes are relatively standard for similar analyses on wild animal populations (e.g. sample sizes are between 306–576 Soay sheep (Coltman *et al.* 2001) and 333–634 red deer (Clements *et al.* 2011) for some heritability estimates on wild mammal populations).

### Associations between strongyle infection and physiological traits

Previous work on this study population has also found associations between EPG and other key components of individual phenotypes, specifically survival rates and body size (Thumbi *et al.* 2013a, b). Thus strongyle EPG has a major impact on life history in this population. We have added to this information the contribution of the different components of variance in each of these traits, and the observation that birth weight predicts subsequent worm infection.

Calves with a higher strongyle EPG tended to have lower mean EO, WBC, RBC and TSP than those with fewer eggs: these associations applied both to average values across all observations on a calf (the ‘individual-level’ covariances in Table 4), and within each visit (‘residual’ covariances in Table 4). Some strongyle species, such as *H. placei*, are important causes of anaemia in cattle (Kaufmann *et al.* 1992). Since anaemia is defined as an erythrocyte count, haemoglobin concentration or packed cell volume below the reference value for that species (Jain, 1993), it is expected that RBC will decrease in association with strongyle infection, as we observed in this study. Furthermore, as some strongyles such as *H. placei* are blood sucking parasites, a reduction in all blood parameters at the same time is likely to be due to total blood loss in calves with high burdens. The loss in TSP will probably also contribute to the reduction in weight. Meanwhile, the negative association between EO and strongyle EPG could be explained by EO having been implicated in the resistance to infection in ruminants. For example, Bricarello *et al.* (2007) found a negative association between nematode FEC and blood eosinophil counts in Nelore-breed cattle.

Calves that were lighter weight at less than 1 week old had a higher strongyle EPG than heavier calves when they are aged 16–51 weeks old. In a study of humans, Raqib *et al.* (2007) observed altered immune function in low birth weight babies which may increase vulnerability to infection later in life. Alternatively, the association could be generated by correlations of both early weight and subsequent strongyle infection with some other unmeasured aspect of individual condition, without requiring any causal component. It is also possible that lighter calves may be eating less and therefore might be expected to have lower intensities of infection, due to sampling fewer areas, but we observe the opposite direction of effect, with lighter calves having a higher strongyle EPG. However we did not monitor the calves’ consumption of food during the study so cannot investigate this further.

### Concluding remarks

To conclude, in this study we have used relationship matrices reconstructed from SNP genotypes to demonstrate evidence for heritable variation in strongyle EPG in EASZ. We also found significant additive genetic variation in WBC. All additional traits investigated showed negative phenotypic covariances with strongyle EPG throughout the first year: high strongyle EPG was associated with low WBC, RBC, TSP, and EO. Weight at 1 week old was significantly associated with strongyle EPG at 16–51 weeks: smaller calves had a higher strongyle EPG later in life. Our results indicate that additive genetic variation in strongyle EPG is present in this population, and that strongyle EPG is associated with variation in other important variables. Further investigation is needed to understand the physiological mechanisms of the interactions between strongyle EPG and haematological parameters that allow EASZ calves to tolerate a high strongyle EPG.

## Supplementary Material

To view supplementary material for this article, please visit http://dx.doi.org/10.1017/S0031182014001498

Supplementary Material

## Figures and Tables

**Table 1:** Variance components (± s.e.) for all traits considered in univariate repeated measures models which include calf age and sex as fixed effects and *V_A_*, *V_PE_* and *VSL* as a random effect

Trait	Number of observations	Mean (±s.d.)	*V_SL_*	*V_A_*	*V_PE_*	*V_RES_*	*h*^2^ (%)	*V_PE_*/*V_P_* (%)	*r*^2^ (%)	Sex effect estimate
Strongyle EPG	4032	542·72 ± 15·79	0·06 ± 0·03	0·45 ± 0·23	0·08 ± 0·22	1·30 ± 0·03	23·92 ± 11·83	4·32 ± 11·53	31·44 ± 2·16	0·23 ± 0·08
WBC (× 10^3^ *μ*L^− 1^)	4693	11·08 ± 3·44	0·22 ± 0·15	3·15 ± 1·22	0·86 ± 1·17	7·17 ± 0·16	27·63 ± 10·56	7·54 ± 10·28	37·17 ± 1·97	− 0·15 ± 0·21
RBC (× 10^6^ *μ*L^− 1^)	4281	8·79 ± 2·03	0·12 ± 0·06	0·57 ± 0·41	0·60 ± 0·04	1·95 ± 0·04	17·60 ± 12·46	18·50 ± 12·36	39·68 ± 2·10	− 0·26 ± 0·11
TSP (g dL^− 1^)	4721	7·98 ± 0·90	2·56 × 10^−3^ ± 3·15 × 10^−3^	0·07 ± 0·04	0·03 ± 0·04	0·38 ± 0·01	14·50 ± 8·20	7·05 ± 8·09	22·08 ± 1·63	− 0·04 ± 0·04
EO	3690	0·14 ± 0·14	1·71e^− 4^ ± 1·12e^− 4^	9·44e^− 5^ ± 8·78e^− 4^	1·74e^− 3^ ± 9·14e^− 4^	1·57e^− 2^ ± 3·89e^− 4^	0·53 ± 4·97	9·84 ± 5·15	11·34 ± 1·44	− 0·01 ± 0·01
Weight	3338	1·55 ± 0.18	4·19e^− 4^ ± 2·41e^− 4^	1·91e^− 3^ ± 1·87e^− 3^	4·46e^− 3^ ± 1·87e^− 3^	2·92e^− 3^ ± 7·69e^− 5^	19·64 ± 19·22	45·93 ± 19·06	69·89 ± 1·67	0·02 ± 0·01

The proportion of total variance (*V_P_*) explained by the permanent environment variance (*V_PE_*) is also presented. The total number of calves for each trait is 446. EO, transformed EO (× 10^3^ *μ*L^− 1^, log_10_(EO + 1)); weight, transformed body weight (kg, log_10_(weight)); *V_SL_*, sublocation variance; *V_A_*, additive genetic variance; *V_PE_*, permanent environment variance; *V_RES_*, residual variance; *h*^2^, heritability; *V_PE_*/*V_P_* (%), proportion of the total phenotypic variance explained by the permanent environment variance expressed as a percentage; *r*^2^, repeatability; sex effect estimate, the effect estimate of being male.

**Table 2:** The effect of a high or low strongyle EPG at a given age on the trait of interest, using univariate animal models

Trait	WBC				RBC				TSP			
	Estimate	s.e.	*F*	*P* value	Estimate	s.e.	F	*P* value	Estimate	s.e.	F	*P* value

Intercept	9·676	0·217	8049·50	< 0·001	10·560	0·124	10999·24	< 0·001	9·690	0·041	200000·00	< 0·001
High strongyle EPG	− 0·471	0·102	0·35	0·550	− 0·362	0·054	236·11	< 0·001	− 0·090	0·023	646·29	< 0·001
Sex (effect of being male)	− 0·046	0·210	0·05	0·819	− 0·224	0·111	4·04	0·047	− 0·034	0·036	0·88	0·349
Calf age (11 factor levels)	NA	NA	25·94	< 0·001	NA	NA	184·00	< 0·001	NA	NA	269·40	< 0·001

Trait	EO						Weight					
	Estimate		s.e.	*F*		*P* value	Estimate		s.e.	*F*		*P* value

Intercept	0·055		0·009	1166·54		< 0·001	1·267		0·008	72274·08		< 0·001
High strongyle EPG	− 0·007		0·005	10·96		0·001	− 0·015		0·003	3579·83		< 0·001
Sex (effect of being male)	− 0·009		0·006	2·22		0·14	0·018		0·008	5·44		0·021
Calf age (11 factor levels)	NA		NA	37·15		< 0·001	NA		NA	2682·21		< 0·001

A high EPG is defined as being above the median strongyle EPG whilst a low EPG is defined as being below the median strongyle EPG. The median is the overall median taken across all visits. The significance is given by the Wald *F* statistic. NA, not applicable, as multiple factor level estimates are not reported.

**Table 3:** Association between strongyle EPG in older calves (aged 16–51 weeks old) and the calf ’s weight at the recruitment visit (calf aged <1 week), using a univariate animal model.

Variable	Estimate	s.e.	*P* value
Calf weight at recruitment (kg)	−0·030	0·012	0·011
Calf sex (effect of being male)	0·276	0·087	0·006
Calf age (7 factor levels)	NA	NA	0·185

**Table 4:** Covariance/variance/correlation matrix for the between-individual and residual (within-individual) level variance between strongyle EPG and trait

	Strongyle EPG	WBC	RBC	TSP	EO	Body weight
Individual level variance						
Strongyle EPG	*0·620 (0·065)*	− 0·120 (0·068)	− 0·386 (0·062)	− 0·192 (0·075)	− 0·140 (0·098)	− 0·182 (0·060)
WBC	− 0·204 (0·118)	*4·678 (0·426)*	0·133 (0·064)	0·284 (0·068)	0·468 (0·079)	0·280 (0·054)
RBC	− 0·355 (0·067)	0·336 (0·168)	*1·365 (0·128)*	0·251 (0·069)	0·138 (0·092)	0·399 (0·050)
TSP	− 0·046 (0·019)	0·188 (0·049)	0·090 (0·027)	*0·094 (0·011)*	0·030 (0·104)	0·273 (0·061)
EO	− 0·005 (0·004)	0·048 (0·010)	0·008 (0·005)	0·0004 (0·001)	*0·002 (0·0004)*	0·368 (0·077)
Body weight	− 0·013 (0·004)	0·053 (0·011)	0·041 (0·006)	0·007 (0·002)	0·002 (0·0003)	*0·008 (0·001)*
Residual level variance						
Strongyle EPG	*1·124 (0·041)*	− 0·095 (0·025)	− 0·140 (0·025)	− 0·124 (0·025)	− 0·022 (0·026)	− 0·137 (0·025)
WBC	− 0·255 (0·069)	*6·472 (0·239)*	0·345 (0·023)	0·152 (0·025)	0·246 (0·024)	0·126 (0·026)
RBC	− 0·214 (0·040)	1·262 (0·101)	*2·065 (0·046)*	0·265 (0·024)	0·082 (0·026)	0·209 (0·025)
TSP	− 0·067 (0·014)	0·199 (0·034)	0·196 (0·020)	*0·265 (0·010)*	0·032 (0·026)	0·182 (0·025)
EO	− 0·003 (0·003)	0·078 (0·009)	0·015 (0·005)	0·002 (0·002)	*0·016 (0·001)*	0·018 (0·026)
Body weight	− 0·007 (0·001)	0·016 (0·003)	0·015 (0·002)	0·005 (0·001)	0·0001 (0·0002)	*0·002 (0·0001)*

Covariances are shown below the diagonal (in italics), the associated correlations above the diagonal and variances on the diagonal. Standard errors are in brackets. WBC, white blood cell count (− 10^3^ *μ*L^−1^); RBC, red blood cell count (× 10^6^ *μ*L^−1^); TSP, total serum protein (gdL^−1^); EO, transformed absolute eosinophil count (× 10^3^ *μ*L^−1^, log_10_(EO + 1)); body weight, transformed body weight (kg, log_10_(weight)).
