# Supplementary material for: Variation and covariation in strongyle infection in East African shorthorn zebu calves
Source: Parasitology. Author manuscript; Available in PMC 2018 Mar 9. (PMC5844460; doi:10.1017/S0031182014001498)
Supplement: Supplementary Material [file NIHMS76176-supplement-Supplementary_Material.docx]

## SUPPLEMENTARY MATERIALS

#### Impact of different quality control parameters and marker density on heritability estimates

Different SNP quality control checks result in different heritability estimates. A balance is needed to be found between quality control checking and removing too much of the variation from the population. Supplementary Table 1 presents the heritability estimates for strongyle EPG in the univariate animal model as a result of varying the parameters in the quality control checks on the SNP data. All quality control cut-offs used in each section of Supplementary Table 1 produce very similar heritability estimates. However removal of the European taurine introgressed calves produce lower heritability estimates with higher standard errors than models which include the European taurine introgressed calves, this is discussed in more detail in the “Effect of including European taurine introgressed calves in our study population” section of the supplementary materials.

A SNP call rate cut-off = 0.9; individual call rate=0.9, identity by state (IBS) threshold of 0.9 and a Hardy-Weinberg Equilibrium cut-off of 1.00e^-8^ was chosen to build the models within this paper. The minor allele frequency for SNPs was set to 0.005, to include all SNPs where the minor allele count was 5 or more.

To assess the effect of the number of markers on the heritability, we randomly sampled sets of 25, 50 and 75% of all autosomal markers corresponding to 10530, 21060, 31589 autosomal SNPs and examined their effect upon the heritability estimates of strongyle EPG. We show that the estimate of heritability of strongyle EPG increased with increasing marker density, and it was only in the model involving 100% of the markers which passed quality control that estimates were significantly different from zero (Supplementary Table 2).

Supplementary Table 1: Effect of changing the quality control cut-offs on the heritability of strongyle EPG using a univariate animal model with a negative binomial errors. The estimate is followed by its standard error in brackets. The impact of quality control on a study population with European taurine introgressed calves either a) included or b) excluded is also presented. See section of the supplementary materials called “Effect of including European taurine introgressed calves in our study population” for an explanation of European taurine introgressed calves.

| 1. All calves | |  |  |  |  |  |  |
| --- | --- | --- | --- | --- | --- | --- | --- |
| **SNP call rate** | **Individual call rate** | **Minor allele frequency** | **P value of Hardy-Weinberg Equilibrium** | **Identity by state cut-off** | **Number of alive individuals left after quality control** | **Number of markers left after quality control** | **Heritability (%)** |
| 0.9 | 0.9 | 0.005 | 1.00E-08 | 0.9 | 446 | 42119 | 23.92 (11.83) |
| 0.9 | 0.9 | 0.001 | 1.00E-08 | 0.9 | 446 | 45241 | 27.12 (11.89) |
| 0.99 | 0.99 | 0.01 | 1.00E-08 | 0.99 | 440 | 36416 | 22.31 (12.16) |

| b)      Without European taurine introgressed calves | | | | | | | |  |
| --- | --- | --- | --- | --- | --- | --- | --- | --- |
| **SNP call rate** | **Individual call rate** | **Minor allele frequency** | **P value of Hardy-Weinberg Equilibrium** | **Identity by state cut-off** | **Number of alive individuals left after quality control** | **Number of markers left after quality control** | **Heritability (%)** | |
| 0.9 | 0.9 | 0.005 | 1.00E-08 | 0.9 | 353 | 40381 | 13.25 (13.37) | |
| 0.9 | 0.9 | 0.001 | 1.00E-08 | 0.9 | 353 | 43460 | 14.30 (13.99) | |
| 0.99 | 0.99 | 0.01 | 1.00E-08 | 0.99 | 348 | 35678 | 9.18 (13.23) | |

Supplementary Table 2: Effect of the number of markers on the heritability of strongyle EPG. Estimates are followed by their standard errors in brackets.

| **Percentage of Autosomal Markers Sampled** | **Number of Markers Sampled** | **Heritability (%)** |
| --- | --- | --- |
| 25 | 10530 | 9.27 (9.83) |
| 50 | 21060 | 17.58 (10.81) |
| 75 | 31589 | 19.43 (11.64) |
| 100 | 42119 | 23.92 (11.83) |

| 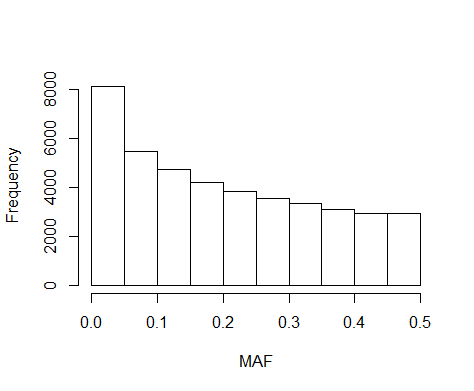 | 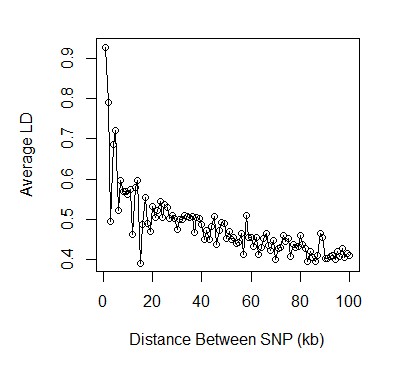 |
| --- | --- |

Supplementary Figure 1: a) The distribution of minor allele frequencies (MAF) at SNP markers and b) The average linkage disequilibrium (LD) in 1kb bins between pairs of SNPs plotted against the physical distance between SNPs in the pair.

#### Effect of including European taurine introgressed calves in our study population

Previous research by Mbole-Kariuki *et al*. (2014) has indicated that there is evidence for European taurine introgression being present in the study population. Mbole-Kariuki *et al*. (2014) used the Ward clustering method (Ward 1963) to identify clusters in the distribution of European taurine ancestry. The first of these categories included calves with ≥12.5% European taurine background (representing animals with “substantial” European introgression, n = 29); the second included calves with between 1.56% to 12.5% European taurine introgression (representing the “moderate” European taurine introgressed, n = 94) and the third category included calves with less than ≤1.56% European taurine background (representing the “non-European introgressed”, n = 425), (Mbole-Kariuki *et al.* 2014). Principal component analysis showed that the main structuring was between the taurine and zebu breeds and the least genetic differentiation was observed between the European taurine breeds (Mbole-Kariuki *et al.* 2014). Therefore it was important to assess the effect of including European taurine (ET) introgressed cattle on our heritability estimates.

In order to assess the effect of including ET introgressed cattle on the heritability estimates, the heritability of strongyle EPG with and without the introgressed calves included was calculated (Supplementary Table 1). Removal of the ‘introgressed’ calves from the study resulted in a lower heritability estimate and larger standard errors (with ET introgressed calves included h^2^ = 23.9%, SE = 11.8%, N calves= 446; with ET introgressed calves excluded h^2^ = 13.3%, SE = 13.4%, N calves= 353). The decrease in heritability is likely to be due to the European introgressed calves having a higher genetic variance whilst the larger standards errors are possibly due to a decrease in sample size. Consequently, we choose to include the ET introgressed calves in our study population to maximise the same size and amount of variation in the population.

The effect of including ET introgression can also be accounted for by including ET introgression as a continuous fixed effect in our models. The genomic relationship matrix and model structures for these analyses are the same as the one used in the main section of this paper, and so the only difference is the additional ET term. The impact of including ET as a fixed effect in all our analyses is presented in Supplementary Table 3.

Supplementary Table 3 shows that the heritability estimates for all traits investigated are very similar to those from the models without ET introgression fitted explicitly. ET has little effect on any of results and its effect estimate does not significantly differ from zero. For example the heritability of Strongyle EPG without ET fitted as a fixed effect equals 23.9% (SE = 11.8, Supplementary Table 1) and with ET fitted as a fixed affect the heritability of strongyle EPG equals 25.7% (SE = 11.9, Supplementary Table 3). Furthermore, since this paper is interested in investigating the causes of variance, it is important to include as much variability in our population as possible.

Supplementary Table 3: Impact of including European Taurine introgression as a continuous fixed effect in the univariate animal model with negative binomial errors distribution for strongyle EPG and the univariate animal models with Gaussian errors for the physiological traits. The rest of the model has exactly the same structure as that used in Table 1. The genomic relationship matrix includes all calves, both introgressed and not. (log_10_(EO + 1)) = transformed absolute eosinophil count (x10^3^/μL); log_10_(Weight) = transformed body weight (kg); V_SL_ = sublocation variance; V_A_ = additive genetic variance; V_PE_ = permanent environment variance; V_RES_ = residual variance; h^2^ = heritability; r^2^ = repeatability.

| **Trait** | **V_SL_** | **V_A_** | **V_PE_** | **V_RES_** | **h^2^ (%)** | **r^2^ (%)** | **ET effect estimate** | **ET effect estimate P value** |
| --- | --- | --- | --- | --- | --- | --- | --- | --- |
| Strongyle EPG | 0.06 ± 0.03 | 0.49 ± 0.23 | 0.05 ± 0.22 | 1.3 ± 0.03 | 25.74 ± 11.85 | 31.52 ± 2.16 | 0.35 ± 1.93 | 0.86 |
| White Blood Cell Count (x10^3^/μL) | 0.21 ± 0.14 | 3.07 ± 1.25 | 0.93 ± 1.2 | 7.17 ± 0.16 | 26.96 ± 10.83 | 36.98 ± 1.97 | -7.31 ± 4.85 | 0.14 |
| Red Blood Cell Count (x10^6^/μL) | 0.12 ± 0.06 | 0.62 ± 0.41 | 0.55 ± 0.4 | 1.95 ± 0.04 | 19.15 ± 12.56 | 39.72 ± 2.11 | -1.91 ± 2.4 | 0.44 |
| Total Serum Protein (g/dL) | 0.002 ± 0.003 | 0.07 ± 0.04 | 0.03 ± 0.04 | 0.38 ± 0.01 | 15.33 ± 8.25 | 22.09 ± 1.64 | -0.52 ± 0.79 | 0.52 |
| log_10_(EO + 1) | 0.0001 ± 0.0001 | 0.0000 ± 0.0000 | 0.002 ± 0.000 | 0.016 ± 0.000 | 0.00 ± 0.00 | 11.17 ± 1.42 | -0.16 ± 0.09 | 0.06 |
| log_10_(Weight) | 0.0004 ± 0.0002 | 0.0008 ± 0.0021 | 0.0055 ± 0.0021 | 0.0029 ± 0.0001 | 8.57 ± 21.77 | 69.72 ± 1.67 | 0.26 ± 0.13 | 0.10 |

#### Comparison of model assuming Gaussian or negative binomial errors

Previous work of this kind, for example Beraldi *et al.* (2007); Bishop *et al.* (1996); Coltman *et al.* (2001) and Stear *et al.* (1990) all used log transformations in their estimations of the heritability of faecal egg counts in different species. In Supplementary Table 4, we present results of univariate models of strongyle EPG with both Gaussian and negative binomial errors. The Gaussian model uses a log_10_ (Strongyle EPG + 50) transformation. Both methods produce similar estimates of heritability of strongyle EPG (Supplementary Table 4– though, interestingly, the SEs are much larger with the GLMM).

Supplementary Table 4: Results of the univariate animal models for the heritability and variance components of Strongyle EPG across all ages using Generalised Linear Mixed Models (GLMM) fitted with a negative binomial distribution and a Linear Mixed Model (LMM) with a Gaussian distribution in which the strongyle EPG has been log-transformed.

|  | GLMM | | LMM | |
| --- | --- | --- | --- | --- |
|  | Estimate | SE | Estimate | SE |
| Sublocation Variance | 0.061 | 0.031 | 0.006 | 0.003 |
| Permanent Environment Variance | 0.082 | 0.218 | 8.34x10^-8^ | 1.98x10^-9^ |
| Additive Genetic Variance | 0.452 | 0.226 | 0.049 | 0.005 |
| Residual Variance | 1.296 | 0.031 | 0.168 | 0.004 |
| Heritability (%) | 23.92 | 11.83 | 22.09 | 1.79 |

#### Age related trends in the other traits of interest


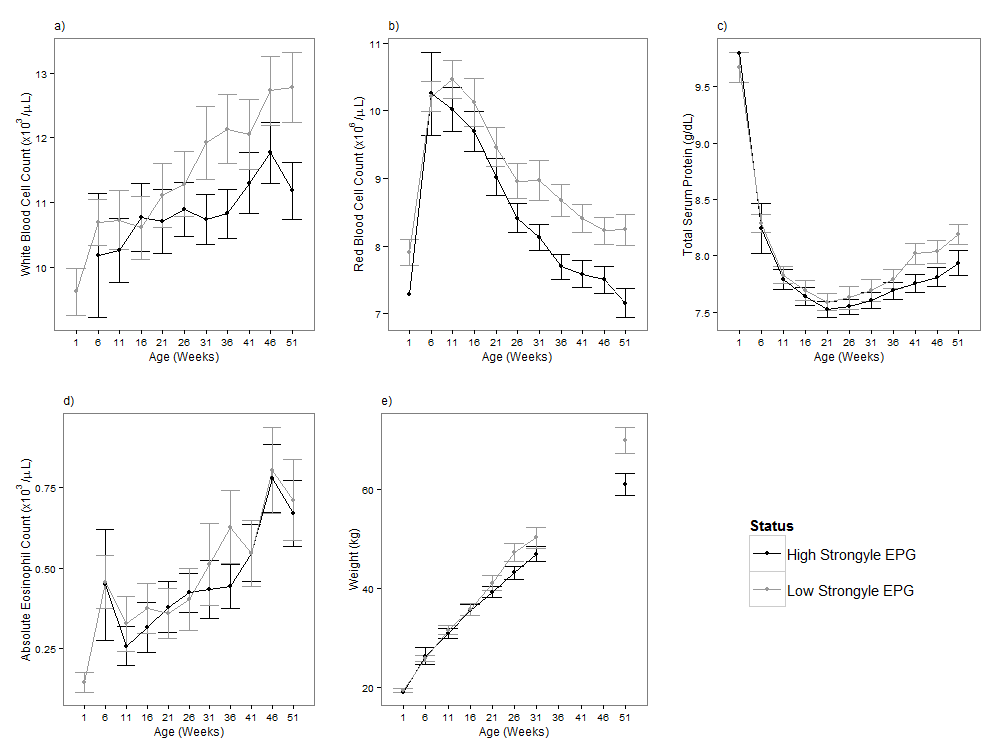


## Supplementary Figure 2: The age-related changes in the other traits for calves with a high (black line) and low (grey line) strongyle EPG. A high strongyle EPG is defined as being above the overall median strongyle EPG and a low EPG is below the median strongyle EPG. The median is the overall median taken across all visits, and is equal to 200 EPG. Error bars represent the 95% confidence intervals. Traits shown are (a) White Blood Cell Count; (b) Red Blood Cell Count; (c) Total Serum Protein; (d) Absolute Eosinophil Count; (e) Body Weight.

## REFERENCES

**Beraldi, D., McRae, A. F., Gratten, J., Pilkington, J. G., Slate, J., Visscher, P. M. and Pemberton, J. M.** (2007). Quantitative trait loci (QTL) mapping of resistance to strongyles and coccidia in the free-living Soay sheep (*Ovis aries*). *International Journal for Parasitology,* **37**, 121-129. doi: 10.1016/j.ijpara.2006.09.007.

**Bishop, S. C., Bairden, K., McKellar, Q. A., Park, M. and Stear, M. J.** (1996). Genetic parameters for faecal egg count following mixed, natural, predominantly *Ostertagia circumcincta* infection and relationships with live weight in young lambs. *Animal Science,* **63**, 423-428.

**Coltman, D. W., Pilkington, J., Kruuk, L. E. B., Wilson, K. and Pemberton, J. M.** (2001). Positive genetic correlation between parasite resistance and body size in a free-living ungulate population. *Evolution,* **55**, 2116-2125. doi: 10.1111/j.0014-3820.2001.tb01326.x.

**Mbole-Kariuki, M. N., Sonstegard, T., Orth, A., Thumbi, S. M., Bronsvoort, B. M. d. C., Kiara, H., Toye, P., Conradie, I., Jennings, A., Coetzer, K., Woolhouse, M. E. J., Hanotte, O. and Tapio, M.** (2014). Genome-wide analysis reveals the ancient and recent admixture history of East African Shorthorn Zebu from Western Kenya. *Heredity*. doi: 10.1038/hdy.2014.31.

**Stear, M. J., Hetzel, D. J. S., Brown, S. C., Gershwin, L. J., Mackinnon, M. J. and Nicholas, F. W.** (1990). The relationships among ecto- and endoparasite levels, class I antigens of the bovine major histocompatibility system, immunoglobulin E levels and weight gain. *Veterinary Parasitology,* **34**, 303-321. doi: 10.1016/0304-4017(90)90077-O.

**Ward, J. H.** (1963). Hierarchical grouping to optimize an objective function. *Journal of the American Statistical Association,* **58**, 236-244. doi: 10.1080/01621459.1963.10500845.
